# Malting quality of seven genotypes of barley grown in Nepal

**DOI:** 10.1002/fsn3.1743

**Published:** 2020-07-19

**Authors:** Pravin Ojha, Nagina Gautam, Ujjwol Subedi, Narayan B. Dhami

**Affiliations:** ^1^ Food Research Division Nepal Agricultural Research Council Lalitpur Nepal; ^2^ Department of Food Technology Padamshree International College Kathmandu Nepal; ^3^ Hill Crop Research Program Nepal Agricultural Research Council Dolakha Nepal

**Keywords:** barley, diastatic power, malting, viscosity, β‐glucan

## Abstract

There has been very limited work on the malting quality of barley grown in Nepal. This work used completely randomized experiment for seven barley genotypes, namely *Xveola‐45*, *Coll#112‐114/Muktinath*, *Xveola‐38*, *Solu uwa*, *NB‐1003/37‐1038*, *NB‐1003/37‐1034*, and *Bonus,* collected from Hill Crop Research Program (*Dolakha*, Nepal) to study the effect of genotypes on the chemical composition and functional properties of barley and malt. Barley was steeped for 24 hr followed by 72 hr germination in room temperature (25 ± 3°C). Germinated barley was dried (45°C/6 hr, 50°C/4 hr, 55°C/8 hr, 70°C/1 hr, 80°C/3 hr) in a cabinet drier. Multistage dried barley was then ground to pass through a 250 µm screen. Among the chemical composition, protein and reducing sugar were affected by genotype (*p* < .05) in barley except for β‐glucan. Functional properties, particularly bulk density, water absorption capacity, oil absorption capacity, and viscosity, were affected by genotype (*p* < .05) in barley, whereas except for density, all the parameters were different (*p* < .05) for malt. The highest diastatic power among all genotypes was recorded for *solu uwa* (329.25 ºDP) followed by *Muktinath* (271.15 ºDP). There was no significant change (*p* < .05) in a protein of all genotypes after malting, whereas β‐glucan and viscosity significantly decreased (*p* < .05) for all genotypes after malting. The remaining parameters for all genotypes were not affected (*p* < .05) by malting. *Solu uwa* had higher enzymatic activity, whereas *Xveola‐38* and *Muktinath* were found to be better for complimentary food preparation.

## Graphical abstract

This work used completely randomized experiments for seven barley genotypes to study the effect of genotypes on the chemical composition and functional properties of malted barley. Among the chemical composition, protein and reducing sugar were affected by genotype (*p* < .05) in barley except for β‐glucan. Functional properties, particularly bulk density, water absorption capacity, oil absorption capacity, and viscosity, were affected by genotype (*p* < .05) in barley, whereas except for density, all the parameters were different (*p* < .05) for malted barley.

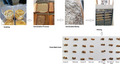

## 1 INTRODUCTION

Barley, one of the earliest cultivated grain in a wide variety of climate across many geographical regions (Newman & Newman, 2006), is industrially malted and brewed for both food and feed purposes. On a different basis, barley can be classified as two‐row (*Hordeum distichum*) or six‐row (*Hordeum vulgare*); hull or hull‐less barley (naked barley); winter and spring barley (Baik & Ullrich, 2008; Vasan, Mani, & Boora, 2014; Zhou, 2009). This crop stands as the fourth main important crop globally and as the fifth major crop in Nepal after rice, wheat, maize, and millet, with production and yield of 30,510 Mt and 1.115 Mt/ha, respectively (MoAD, 2017). In Nepal, barley is utilized in food and nonfood purpose. Mostly in the hilly areas of Nepal. It is used to prepare food viz. fermented alcoholic beverages and other food items. However, nonfood use (worshiping during religious rituals) is limited.

Malting, a combined process of controlled germination and drying, is done to obtain a desirable physical and biochemical change within the grain by the subsequent development of hydrolytic enzymes (Dewar, 2003; Gupta, Abu‐Ghannam, & Gallaghar, 2010; Osman, Coverdale, Onley‐Watson, Bell, & Healy, 2003). The main processes carried out to assure the required biochemical changes include moistening of grain from 12% to 40% This is followed by germination to synthesize enzymes and endosperm hydrolysis. Finally, a kilning process is carried out to stop the previous enzymatic activity (Gupta et al., 2010). In the case of Nepal, germination of barley is restricted to cultural practices, where germinated grass of barley is considered as holy leaves and offered to the loved ones by respected elders as a blessing of goddess *Durga* during *Dashain* (a Hindu festive). They accept it in the head and discard it as a waste when it is dried.

Barley is the most genetically diverse nature of the crop (Baik & Ullrich, 2008), its flour quality is different from genotypes and will affect the product characteristics. The functional properties of flour are interrelated with the chemical composition (mainly protein and carbohydrate). Chandra, Singh, and Kumari (2015) stated that the quality of flour is also associated with the structure, molecular confirmation, and physiochemical properties of crops. Thus, the quality must be assessed in terms of the functional property before using it in a product.

Breeders are always looking for the variety with excellent agronomic performance along with better malting characteristics. There had been a work to improve the genotypes of barley since 1973/74 as National Hill Crops Improvement Program and finally changed to Hill Crop Research Program after the establishment of NARC in 1991 (HCRP, 2018). Recently, *Bonus* and *Solu uwa* are the typical variety of barley and naked barley, respectively, released by NARC, which had acquired good responses from the farmers of Nepal, due to high yield, and disease resistance.

However, the selection was made based on agronomic performances like yield, disease‐resistant, and input required. So the study will assist in the selection of genotype during potential new product development. The current trend of research is only focused on the agronomic performances of barley in Nepal. However, limited literature was found in the malting quality and functional properties of Nepalese barley. There is limited industrial processing of barley, but rather the use is concentrated in household applications and feed purposes. Hence, work is needed to assess the malting quality of barley and to study the chemical and functional properties changes in malt. This study will also document the chemical and functional properties of barley genotypes in Nepal. This work will help to identify the suitable variety with better malting characteristics and provide base data for breeders to improve the quality of the germplasm. Henceforth, this research will be a step forward to identify the industrial potentiality of barley variety available in Nepal.

## 2 MATERIALS AND METHODS

### 2.1 Materials

Seven different types of registered and unregistered genotypes of barley namely *Xveola‐45*, *Coll#112‐114*/*Muktinath*, *Xveola‐38*, *Solu uwa*, *NB‐1003/37‐1038*, *NB‐1003/37‐1034*, and *Bonus* were collected from Hill Crop Research Program, Dolakha, Nepal. *Bonus* and *Solu uwa* are the released variety (taken as control), so the chemical and functional properties of the remaining varieties were compared with them. The maturation time and yield are shown in Table 1 (HCRP, 2018).

**TABLE 1:** Maturation time and yield of barley genotypes

Barley genotypes	*Xveola‐45*	*Muktinath*	*Xveola‐38*	*Solu uwa*	*NB‐1003/37‐1038*	*NB‐1003/37‐1034*	*Bonus*
Yield (t/ha)	4.2	4.34	4.34	1.9	3.39	3.24	3.6
Maturation Day	153	140	140	177	149	149	162

### 2.2 Chemicals

The chemicals used in this study are not limited to DNS (3,5‐dinitrosalicylic acid) (98% purity), and glucose standard as procured from HiMedia Laboratories Pvt. Ltd. India. Iso‐propanol alcohol (2‐propanol, IPA) (99% <purity), and starch were obtained from Merck specialties Pvt. Ltd., India.

### 2.3 Methods

#### 2.3.1 Malting

**FIGURE 1:**
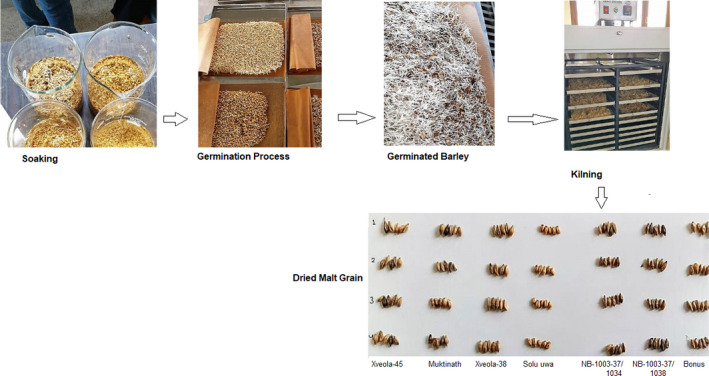
Diagrammatic presentation of preparation of malt grains (*Xveola‐45*, *Coll#112‐114*/*Muktinath*, *Xveola‐38*, *Solu uwa*, *NB‐1003/37‐1038*, *NB‐1003/37‐1034*, *Bonus*)

**FIGURE 2:**
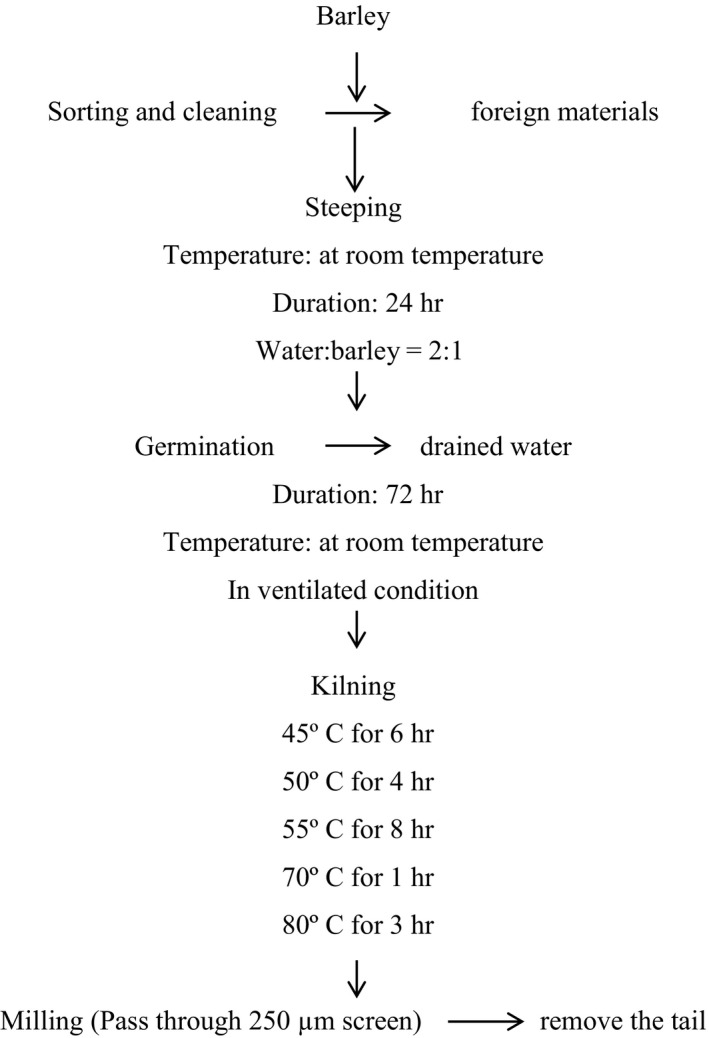
Schematic flow diagram for the preparation of malt from different genotypes of barley found in Nepal

The malting process for the barley was done as described by Bera, Sabikhi, and Singh (2018) with some modifications. The diagrammatic flowchart is shown in Figure 1 while the detailed of the steps for malting is described in Figure 2. After sorting immature grain and foreign materials through winnowing and dry cleaning, barley (1,000 g) was soaked in portable water for 24 hr at room temperature (25 ± 3°C), with draining of water at 12 hr interval. After 24 hr, soaked barley was placed in a blotting paper to reduce extra surface water. Then, it was kept in the previously wetted blotting paper and covered with wetted paper (to maintain RH above 90%). The whole set up was left at room temperature for 72 hr with a periodic sprinkling of water to facilitate germination of the barley. The length of the sprout was around 30 mm after germination.

Multistage drying as suggested by Karki, Mishra, Shrestha, Ojha, and Subedi (2014) was carried out for 22 hr (45°C/6 hr, 50°C/4 hr, 55°C/8 hr, 70°C/1 hr, and 80°C/3 hr) in a cabinet drier until it turns brittle. Dried barley was milled to pass through a 250 µm screen to remove the root (tail). The prepared malt was then packed in an airtight HDPE plastic container until analysis.

Barley was also ground to pass through a 250 µm screen. It was then packed in an airtight HDPE container until analysis.

#### 2.3.2 Analyses

##### Moisture

The moisture content of the barley and malt was determined as described by AOAC method number 930.15 (AOAC, 2005) with a hot air oven at 106°C for about 6 hr until constant weight was obtained.

##### Crude protein

The nitrogen content of barley and malt was determined by the Kjeldahl method as described by AOAC method number 920.152 (AOAC, 2005). The crude protein was determined by multiplying the nitrogen content with a factor 6.25.

##### Reducing sugar

A colorimetric method as per Ranganna (1986) was used to assess the reducing sugar of barley and malt. A dinitro salicylic acid DNS reagent was used, and the absorbance was taken at 510 nm against standard glucose solution.

##### β‐Glucan

The β‐Glucan of barley and malt was determined by the alkali extraction method with slight modification, as reported by Kim and Ryu (2003). The flour sample (2 g) was suspended in 25 ml of sodium carbonate–bicarbonate (pH 10.3) and was stirred vigorously at 45°C for 30 min. The suspension was centrifuged at 1,008 *g* for 30 min. The residue was discarded, and the collected supernatant was heated at 100°C for 10 min to inactivate the enzyme and was subsequently cooled. The cooled supernatant was mixed with an equal amount of 2‐proponal (isopropyl alcohol, IPA). Then, the precipitate was allowed to settle overnight. The precipitate was collected by filtration, and the residue was washed with IPA on a suction filter. Then, the amount of crude β‐glucan is expressed in 100 g of flour.

##### Diastatic power

The diastatic power of malt was determined by Fehling's solution modification method (AOAC Official Method 935.31) as per AOAC (2005). The diastatic power was calculated by using formula 1.

(1) $$\text{Degree Diastatic power}=\left(5000/V\right)\times \left(B/V\right)$$

Where *V* = amount of starch solution required to neutralize Fehling's solution,

*B* = amount of starch solution with added NaOH to neutralize Fehling's solution.

#### 2.3.3 Functional property

##### Packed bulk density

For the packed bulk density of barley flour and malt flour, the method described by Kanpairo, Usawakesmanee, Sirivongpaisal, and Siripongvutikorn (2012) with some modification was used. Ten grams of sample was gently filled into a dried 25 ml graduated cylinder, and then, the cylinder was gently tapped for 25 times. The volume of the flour was recorded from the cylinder, and the packed bulk density was calculated using formula 2.

(2) Packed Bulk density=weight of powder/volume of powder

##### Viscosity

A suspension (10%) of barley and malt in distilled water was prepared, which was then mechanically shaken for 2 hr of the period at room temperature and the viscosity of prepared suspension was measured by using Ostwald U‐tube viscometer taking water as a standard liquid (distilled water in this case) (Nwosu, 2011), and the viscosity was calculated using the formula 3.

(3) $$\cap 1=\left(D1\times t1/D2\times t2\right)\times \cap 2$$

Where ∩_1_ = Viscosity of unknown liquid.

∩_2_ = Viscosity of standard liquid.

D_1_ = Density of unknown liquid.

D_2_ = Density of standard liquid.

T_1_ = time of flow of unknown liquid.

T_2_ = time of flow of standard liquid.

##### Water absorption capacity (WAC)

A method was adapted from Nwosu, Owuamanam, Omeire, and Eke (2014), where one g of barley and malt flour sample was weighted separately in a clean and dry falcon tube followed by the addition of 10 ml distilled water. Tubes were then centrifuged at 448 *g* for 15 min in a laboratory centrifuge. The tube with flour was reweighed after discarding the supernatant. The change in mass based on the initial mass was calculated as the water absorption capacity of the flour sample.

##### Oil absorption capacity (OAC)

For oil absorption capacity, a method was adopted form Onuegbu, Nworah, Essien, Nwosu, and Ojukwu (2013) with slight modification. Sunflower oil (Sp. gr. 0.92) was used to estimate the oil absorption capacity. The method was similar to water absorption capacity, but oil was used instead of water, and centrifugal rotation was carried out for 20 min.

### 2.4 Statistical analysis

The research design was completely randomized experiments for seven genotypes and their malt with triplicate analysis for analytical parameters. IBM SPSS statistics version 20 (IBM corporation) was used for the statistical analysis. One way ANOVA was used to see the effect of genotype at a 5% level of significance for both barley and malt. Tukey's test was carried out to conduct a post hoc test. The effect of malting was tested by *t* test for each genotype individually using SPSS version 20 programming at a 95% level of confidence.

## 3 RESULTS AND DISCUSSION

Seven varieties of barley genotypes were subjected to chemical analysis before and after malting. The functional property of both barley and malt was analyzed.

All the varieties were malted and then analyzed with the same procedure. Hence, the result obtained would be attributed solely dependent on the types of barley used.

### 3.1 Varietal and malting effect on chemical properties of barley

The result obtained for the crude protein (g/100 g), reducing sugar (g/100 g), and β‐glucan (g/100 g) of 7 genotypes of barley and malt is shown in Table 2. The result shows that except for β‐glucan, all other parameters were significantly affected (*p* < .05) by genotypes for barley, while all the above parameters were significantly different for malt (*p* < .05). Table 2 also describes the diastatic activity (ºDP) of malt and found that they significantly differ (*p* < .05).

**TABLE 2:** Chemical composition of barley and malt affected by genotypes

Barley genotype	Barley			Malt			
	Protein (g/100 g)	Reducing sugar (g/100 g)	β‐glucan (g/100 g)	Protein (g/100 g)	Reducing sugar (g/100 g)	β‐glucan (g/100 g)	Diastatic power (ºDP)
*Xveola‐45*	8.17 ± 0.28^bc^	7.3 ± 0.27^c^	9.35 ± 0.4^a^	8.31 ± 0.19^ab^	8.17 ± 0.21^c^	6.23 ± 0.52^ab^	247.14 ± 0.57^d^
*Muktinath*	8.84 ± 0.2^c^	7.1 ± 0.1^c^	9.10 ± 0.61^a^	8.98 ± 0.19^bc^	8.04 ± 0.32^c^	7.09 ± 0.19^b^	271.15 ± 0.57^e^
*Xveola‐38*	7.42 ± 0.14^a^	4.36 ± 0.12^a^	7.74 ± 0.47^a^	7.85 ± 0.27^a^	4.71 ± 0.1^a^	6.13 ± 0.31^ab^	225.02 ± 0.85^c^
*Solu uwa*	8.63 ± 0.1^c^	7.02 ± 0.25^c^	7.73 ± 0.61^a^	9.21 ± 0.43^c^	8.05 ± 0.19^c^	5.50 ± 0.5^a^	329.25 ± 0.53^f^
*NB‐1003/37‐1038*	7.86 ± 0.56^ab^	7.59 ± 0.30^c^	9.57 ± 0.77^a^	8.42 ± 0.2^ab^	7.96 ± 0.19^c^	7.25 ± 0.2^ab^	206.52 ± 0.96^b^
*NB‐1003/37‐1034*	8.53 ± 0.27^c^	5.61 ± 0.1^b^	9.46 ± 0.2^a^	8.58 ± 0.2^ab^	5.9 ± 0.15^b^	8.4 ± 0.27^c^	170.30 ± 0.08^a^
*Bonus*	8.58 ± 0.28^c^	7.77 ± 0.1^c^	9.23 ± 0.52^a^	9.34 ± 0.20^c^	8.41 ± 0.1^c^	7.7 ± 0.19^bc^	254.61 ± 0.25^d^

Note

Values are the mean ± *SE* of mean obtained from the triplicate data. Different letters in the same column indicate significant differences (*p* ˂ .05). All the data are on dry basis (g/100 g).

**FIGURE 3:**
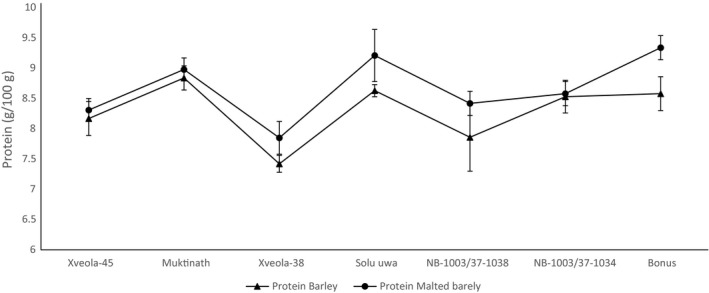
Effect of malting on the protein of barley germplasm

**FIGURE 4:**
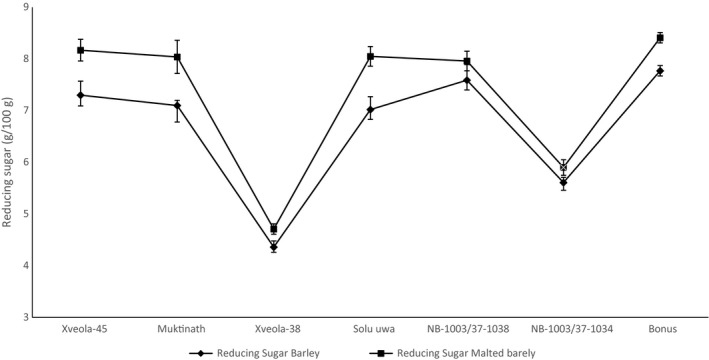
Effect of malting on the reducing sugar of barley germplasm

**FIGURE 5:**
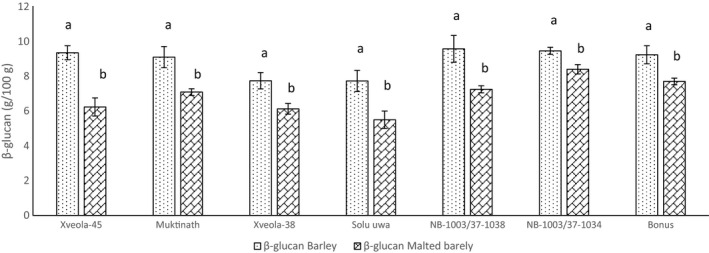
Effect of malting on the β‐glucan of barley germplasm

The effect of malting on the protein (%), reducing sugar (%), and β‐glucan (%) is shown in Figure 3, 4, 5, respectively.

#### 3.1.1 Protein

The protein content of *Xveola‐45* (8.17*)*, *Muktinath* (8.84), and *NB‐1003/37‐1034* (8.53) was comparable to *Bonus* (8.58) and *Solu Uwa* (8.63) in barley. All the variety except *Muktinath* variety had a significantly lower (*p* < .05) protein content than control in malted barley.

Makeri, Nkama, and Badau (2013) reported protein range (%) in five improved varieties of five Nigerian barley varieties ranged from 10 to 12 and 11.8–13.1 for germinated barley. Singkhornart and Ryu (2011) reported protein (%) 8.94 and 9.41–10.22 in barley and germinated barley affected by soaking time and germination temperature. Alijošius et al. (2016) found a protein (%) of 12 varieties of barley (spring and winter) in the range of 10.35–12.38. Choe and Youn (2005) reported protein content of three varieties of barley in the range of 8.07–9.78 grown in Korea. The high percentage of protein in barley produces a beer with haze (Devolli, Dara, Stafasani, Shahinasi, and Kodra, 2018). The protein content of variety depends upon the genotype, its maturity time, and nitrogen application (Eagles, Bedggood, Panozzo, & Martin, 1995). The inference can be drawn that genotype variation affects the protein content of barley and their respective malt.

There was no significant change (*p* < .05) in malt compared to barley for individual genotype. Makeri et al. (2013) reported increase protein in barley after malting. According to Fox et al. (2007), the variation of protein content in the malt is due to the protein content of barley, the method of malting, and highly dependent upon the proteinase activity during malting barley. The protein content increased due to the release of more soluble free amino acid; however, it may decrease if germination time and temperature are increased due to migration of amino acid in sprout (Benincasa, Falcinelli, Lutts, Stagnari, & Galieni, 2019). The reason for no significant change in protein might be due to no change in nitrogen content after malting since protein is calculated based on nitrogen by the Kjeldahl nitrogen method.

#### 3.1.2 Reducing sugar

In barley and malt, the reducing sugar of *Xveola‐45*, *Muktinath*, and NB‐1003/37‐1038 was comparable to *Bonus* and *Solu uwa*, whereas the value for other varieties was significantly lower (*p* < .05) than control. As published by Singkhornart and Ryu (2011), the reducing sugar of barley was found to be 8%, and 12.5%–15.8% in the germinated barley affected by soaking time and germination temperature.

Reducing sugar significantly increased (*p* < .05) for *Xveola‐45*, *Muktinath*, *Solu uwa,* and *Bonus*, whereas there was no change in reducing sugar of *Xveola‐38*, *NB‐1003/37‐1038*, and *NB‐1003/37‐1034* after malting. The significant increment in reducing sugar is observed in the variety with high diastatic activity. The partial dissolution of carbohydrates from the cell wall and hydrolysis of starch increased the reducing sugar in malt (Jamar, du Jardin, & Fauconnier, 2011). Malt extract represents reducing sugar, which is an important parameter for brewing purposes (Aynter, 1999). Genotype variation and malting affect the reducing sugar of barley.

#### 3.1.3 β‐glucan

In barley, β‐glucan was found to be similar in all varieties, whereas in malt, the β‐glucan of *Muktinath* and *NB‐1003/37‐1034* was significantly greater than *Solu uwa* (5.5), but the remaining test varieties were similar to control. The β‐glucan of *Bonus* was also significantly higher (*p* < .05) than *Solu uwa*. The low β‐glucan in *Solu uwa* is because of hull‐less variety. The β‐glucan of all the varieties were significantly decreased (*p* < .05) after malting.

Nishantha et al. (2018) reported β‐glucan of 32 different varieties of barley in the range of 3.26%–7.67%. The β‐glucan of 12 varieties of barley (spring and winter) was reported to be 1.09%–3.95% grown in Lithuanian (Alijošius et al., 2016). Bamforth and Martin (1981) reported β‐glucan of eight varieties of barley and malt on the range of 2.64%–7.28% and depend upon variety, climatic conditions, and soil. He reported a 2%–4% reduction in β‐glucan after malting. Bourne, Powlesland, and Wheeler (1982) found β‐glucan in the barley collected from different locations in the range of 2.7%–4%, and similarly in the malt, the range was 0.18–1.53, and revealed that genotype variation affects the β‐glucan content of barley. Wang, Zhang, Chen, and Wu (2004) reported a more than 80% reduction in β‐glucan due to β‐glucanase activity after malting in all the eight barley cultivars and concluded that β‐glucan content depends both on the cultivar and location. The difference of the result with reported literature might be due to different assay procedures for β‐glucan (Henry & Blakeney, 1986).

The result suggests that increased enzymatic activity results in more reduction in β‐glucan content. Barley with low β‐glucan is suitable for malting and brewing purpose, while a high amount of β‐glucan has a significant contribution to health (Nishantha et al., 2018). The result depicts that β‐glucan is reduced by malting but genotype variation did not have any effect in β‐glucan content in barley.

#### 3.1.4 Diastatic power

The diastatic power of all the varieties was significantly different (*p* < .05). The highest diastatic power was found in *Muktinath* (271.15 ºDP) after *Solu uwa* (329.25 ºDP). The diastatic power of other varieties was significantly less (*p* < .05) than *Bonus* (254.61) and *Solu uwa*. The diastatic power of four varieties of barley was found in the range of 115–142 ºDP (Bera et al., 2018). Diastatic power of malt is combined activities of α‐amylase, β‐amylase, limit dextrinase, and α‐glucosidase (Evans, Li, & Eglinton, 2009). As reviewed by Bera et al. (2018), the variation in diastatic property of malt depends on the genotype, method of preparation of malt, and the process of malting. Gibson, Solah, Holmes, and Taylor (1995) reported that the diastatic power of barley was affected by the cultivar.

The overall composition of barley is affected by genotypes, growing environment (Fox et al., 2007). Tamm, Jansone, Zute, and Jakobsone (2015) revealed that nitrogen supply, maturation date, yield affect the quality traits like protein, carbohydrate, and β‐glucan of barley.

#### 3.1.5 Varietal and malting effect on functional properties of barley

The functional property namely density (g/ml), water absorption capacity (WAI) (%), oil absorption capacity (OAI) (%), and viscosity (cp) before and after malting was studied, shown in Table 3.

**TABLE 3:** Functional quality of barley and malt affected by genotypes

Barley genotype	Barley				Malt			
	BD (g/ml)	V (cp)	WAC%	OAC%	BD (g/ml)	V(cp)	WAC%	OAC%
*Xveola‐45*	0.67 ± 0.04^b^	2.05 ± 0.12^a^	113.98 ± 2.52^c^	97.43 ± 0.43^d^	0.50 ± 0.02^a^	1.59 ± 0.11^bc^	115.29 ± 0.49^b^	99.73 ± 0.87^e^
*Muktinath*	0.78 ± 0.04^c^	2.44 ± 0.13^b^	93.91 ± 0.14^b^	84.13 ± 1.15^ab^	0.51 ± 0.02^a^	1.08 ± 0.11^a^	94.30 ± 1.51^a^	88.60 ± 0.38^c^
*Xveola‐38*	0.66 ± 0.03^b^	2.27 ± 0.12^ab^	122.40 ± 1.89^d^	93.02 ± 0.73^c^	0.51 ± 0.02^a^	1.54 ± 0.11^b^	127.44 ± 0.88^c^	95.37 ± 0.43^d^
*Solu uwa*	0.61 ± 0.03^b^	2.47 ± 0.15^b^	92.05 ± 1.29^b^	85.27 ± 0.48^b^	0.50 ± 0.04^a^	1.75 ± 0.11^c^	116.24 ± 1.16^b^	87.91 ± 1.31^ab^
*NB‐1003/37‐1038*	0.63 ± 0.05^b^	2.11 ± 0.1^a^	80.14 ± 0.5^a^	91.39 ± 0.73^c^	0.50 ± 0.01^a^	1.58 ± 0.12^bc^	93.04 ± 2.3^a^	95.27 ± 0.40^bc^
*NB‐1003/37‐1034*	0.56 ± 0.01^a^	2.18 ± 0.11^a^	105.03 ± 0.57^c^	81.14 ± 0.47^a^	0.51 ± 0.01^a^	1.83 ± 0.1^c^	114.90 ± 3.67^b^	81.95 ± 0.35^a^
*Bonus*	0.63 ± 0.03^b^	2.15 ± 0.09^a^	94.41 ± 2.02^b^	81.63 ± 0.41^a^	0.53 ± 0.01^a^	1.76 ± 0.1^c^	95.52 ± 0.26^a^	85.28 ± 0.07^b^

Note

Values are the mean ± *SE* of mean obtained from the triplicate data. Different letters in the same column indicate significant differences (*p* ˂ .05).

Abbreviations: D, density; OAC, oil absorption capacity; V, viscosity; WAC, water absorption capacity.

The effect of malting on density and viscosity of barley genotypes are shown in Table 4. The effect of malting on the water absorption capacity (WAC%) and oil absorption capacity (OAC%) of seven genotypes of barley is shown in Table 5.

**TABLE 4:** Effect of malting on bulk density and viscosity of barley

Barley genotype	Bulk density (g/ml)		Viscosity (cp)	
	Barely	Malt	Barely	Malt
*Xveola‐45*	0.67 ± 0.04^a^	0.50 ± 0.01^b^	2.05 ± 0.12^a^	1.59 ± 0.11^b^
*Muktinath*	0.78 ± 0.04^a^	0.51 ± 0.01^b^	2.44 ± 0.13^a^	1.08 ± 0.11^b^
*Xveola‐38*	0.66 ± 0.03^a^	0.51 ± 0.01^b^	2.27 ± 0.12^a^	1.54 ± 0.11^b^
*Solu uwa*	0.61 ± 0.03^a^	0.50 ± 0.04^a^	2.47 ± 0.15^a^	1.75 ± 0.11^b^
*NB‐1003/37‐1038*	0.63 ± 0.05^a^	0.50 ± 0.01^b^	2.11 ± 0.16^a^	1.58 ± 0.12^b^
*NB‐1003/37‐1034*	0.56 ± 0.01^a^	0.51 ± 0.01^a^	2.18 ± 0.17^a^	1.83 ± 0.1^b^
*Bonus*	0.63 ± 0.03^a^	0.53 ± 0.01^b^	2.15 ± 0.14^a^	1.76 ± 0.1^b^

Note

Values are the mean ± *SE* of mean obtained from the triplicate data. Different letter in the row in the same section indicates significantly different at *p* < .05.

**TABLE 5:** Effect of malting on water absorption capacity (WAC) and oil absorption capacity (OAC) of barley

Barley genotype	WAC%		OAC%	
	Barely	Malt	Barely	Malt
*Xveola‐45*	113.98 ± 2.52^a^	115.29 ± 0.49^a^	97.43 ± 0.43^a^	99.73 ± 0.87^a^
*Muktinath*	94.30 ± 1.51^a^	93.91 ± 0.14^a^	84.13 ± 1.15^a^	88.6 ± 0.38^b^
*Xveola‐38*	122.40 ± 1.89^a^	127.44 ± 0.88^a^	93.02 ± 0.73^a^	95.37 ± 0.43^b^
*Solu uwa*	92.05 ± 1.29^a^	116.24 ± 1.16^b^	85.27 ± 0.48^a^	87.91 ± 1.31^a^
*NB‐1003/37‐1038*	80.14 ± 0.5^a^	93.04 ± 2.34^b^	91.39 ± 0.73^a^	95.27 ± 0.40^b^
*NB‐1003/37‐1034*	105.03 ± 0.57^a^	114.90 ± 3.67^b^	81.14 ± 0.47^a^	81.95 ± 0.35^a^
*Bonus*	94.41 ± 2.02^a^	95.52 ± 0.26^a^	81.63 ± 0.41^a^	85.28 ± 0.07^b^

Note

Values are the mean ± *SE* of mean obtained from the triplicate data. Different letter in the row in the same section indicates significantly different at *p* < .05.

#### 3.1.6 Bulk density and viscosity

The bulk density (g/ml) of *Muktinath* (0.78) was significantly greater (*p* < .05) than *Bonus* (0.53) and *Solu uwa* (0.61) for barley, whereas the bulk density of *Xveola‐45*, *Xveola‐38*, and *NB‐1003/37‐1038* was comparable to *Bonus* and *Solu uwa*. Genotype variation did not significantly affect the bulk density of malt. The viscosity (cp) of *Muktinath* (2.44) was significantly greater (*p* < .05) than *Bonus* (2.15) and similar to *Solu uwa* (2.47). However, the viscosity of *Solu* uwa was significantly greater (*p* < .05) than *Xveola‐45*, *NB‐1003/37‐1038*, and *NB‐1003/37‐1034*. In malt, the viscosity of *Muktinath* and *Xveola‐38* was significantly less (*p* < .05) than control varieties.

Hamdani et al. (2014) reported the bulk density of barley in the range of 0.5–0.82 g/ml. Pordesimo, Onwulata, and Carvalho (2009) found bulk density 0.511 g/ml and tapped bulk density 0.684 g/ml of barley flour. Bulk density of flour depends upon the size, moisture content, and chemical components (Barbosa‐Cánovas, Ortega‐Rivas, Juliano, & Yan, 2005). Căpriţă and Căpriţă (2012) revealed that viscosity (cp) of barley flour increased from about 3 to 3.82 when left at 100°C from 0 to 15 min. The viscosity of flour is influenced by chemical composition (protein, fat, and β‐glucan) and the interaction between macromolecules (protein, starch, and lipid), molecular weight, temperature, etc. (Crosbie & Ross, 2007).

The density of *Xveola‐45*, *Muktinath*, *Xveola‐38*, *NB‐1003/37‐1038*, and *Bonus* significantly decreased (*p* < .05) after malting, while the viscosity of all the genotypes decreased significantly (*p* < .05) after malting. Ojha et al. (2018) reported a decrease in bulk density and viscosity of sorghum after malting. The decrease in bulk density and viscosity may be due to the breakdown of starch and other high molecular components due to enzymatic activity, and change in surface properties (Chinma et al., 2017; Oti & Akobundu, 2008). Further, protein and lipids can interact with the starch and change its viscosity (Crosbie & Ross, 2007). Low viscosity food is preferred for weaning food, so nutrition dense food can be prepared from germinated barley (Nefale & Mashau, 2018). As reviewed by Mejía, de Francisco, and Bohrer (2020), decreased viscosity can be related to decreased β‐glucan content. Increased reducing sugar and decreased β‐glucan may be responsible for a decrease in density and viscosity of malt. Both reducing sugar and viscosity were reduced by malting and these properties also differ by variety.

*Water Absorption Capacity (WAC) and Oil Absorption Capacity (OAC)*: The WAC (%) of *Xveola‐45*, *Xveola‐38*, and *NB‐1003/37‐1034* were significantly greater (*p* < .05) than control in barley. In malt, the WAC (%) of *Xveola‐38* was 127.44, which was the highest (*p* < .05) among all varieties, whereas the WAC (%) of *Muktinath* (94.3) and *NB‐1003/37‐1038* (93.04) was significantly lower (*p* < .05) than *Solu uwa* (116.24) but similar to *Bonus* (95.52). The OAC (%) of *Xveola‐45*, *Xveola‐38*, and *NB‐1003/37‐1038* was significantly greater (*p* < .05) than *Solu uwa* (85.27) and *Bonus* (81.63), whereas after malting the OAC (%) of *Xveola‐45*, *Muktinath*, and *Xveola‐38* was significantly greater (*p* < .05) than *Solu uwa* (87.91) and *Bonus* (85.28). The water absorption capacity (%) of barley flour was found to be in the range of 70–140 (Chandra et al., 2015). Hussain and Kaul (2018) reported WAI (132.73%) and OAI (180.52%) of barley flour. Abdelazim, Sohair, and Kamel (2019) found WAI (%) and OAI (%) in the range of 140–146 and 78–81 for three varieties of naked barley, respectively. Literature suggests that genotype variation affects the WAC and OAC, which is due to the difference in the chemical composition of flour.

WAC and OAC of the powder depend upon protein concentrations, degree of interactions with amylose, and amylopectin (Butt & Batool, 2010; McWatters, Ouedraogo, Resurreccion, Hung, & Phillips, 2003). The high water absorption index and oil absorption capacity make it useful in various food applications like soups, baked products, meat extenders, as these properties improve the mouthfeel (Onuegbu et al., 2013; Sirivongpaisal, 2008).

The WAC of *Solu uwa*, *NB‐1003/37‐1038*, and *NB‐1003/37‐1034* significantly increased (*p* < .05) after malting, while the OAC of *Muktinath*, *Xveola‐38*, *NB‐1003/37‐1038*, and *Bonus* significantly increased (*p* < .05) after malting.

Ojha et al. (2018) reported an increase in OAC of sorghum flour after malting but did not report a change in WAC after malting. According to Butt and Batool (2010), the observed increase in WAC after malting can be due to different concentrations of protein obtained after malting, their conformational characteristics, and their degree of interaction with water. The WAC and OAC of malted flour generally depend upon the availability of polar amino acids in flours and their association of amylose and amylopectin in the native granules of starch, the number of lipophilic constituents, which was altered after malting (Agrawal, Upadhyay, & Nayak, 2013; McWatters et al., 2003). Agrawal, Upadhyay, and Nayak (2013) reviewed that exposure of hydrophilic and lipophilic constituents of protein is increased by germination and breakdown of polysaccharides facilitate hydration, so WAC and OAC of barley have increased after malting.

## 4 CONCLUSIONS

The chemical and functional properties of barley except β‐glucan were affected by genotype variation, however, in malt, both chemical and functional properties (except density) were affected. β‐glucan and viscosity of all the samples significantly decreased after malting; however, for other parameters, it depends upon the genotype. The significant change in reducing sugar and β‐glucan after malting has a positive relation with diastatic activity. Based on diastatic power, *Solu uwa* and *Muktinath* were found to be best for malting purposes. *Xveola‐45* has high water absorption capacity and oil absorption capacity with a high amount of protein content, while *Muktinath* has a low viscosity. This shows their compatibility with application in composite flour. Further research can be carried out to study the different enzymatic activity in these genotypes and brewing property. Further local drying methods applicable in a rural area can be evaluated in terms of malt quality.

## CONFLICT OF INTEREST

The authors declare that they do not have any conflict of interest.

## ETHICAL APPROVAL

This study does not involve any human or animal testing.

## INFORMED CONSENT

Written informed consent was obtained from all participants.
